# Characterization of Nucleocytoplasmic Shuttling of Pseudorabies Virus Protein UL46

**DOI:** 10.3389/fvets.2020.00484

**Published:** 2020-08-21

**Authors:** Jing-jing Xu, Fei Gao, Ji-qiang Wu, Hao Zheng, Wu Tong, Xue-fei Cheng, Yuting Liu, Haojie Zhu, Xinling Fu, Yifeng Jiang, Liwei Li, Ning Kong, Guoxin Li, Guangzhi Tong

**Affiliations:** ^1^Shanghai Veterinary Research Institute, Chinese Academy of Agricultural Sciences, Shanghai, China; ^2^Jiangsu Co-innovation Center for the Prevention and Control of Important Animal Infectious Disease and Zoonoses, Yangzhou University, Yangzhou, China

**Keywords:** pseudorabies virus, VP11/12, nucleocytoplasmic shuttling, nuclear localization signal, VP16, EP0, STING

## Abstract

Pseudorabies virus (PRV) is the etiological agent of Aujeszky's disease, which has caused severe economic loss in China since its re-emergence in 2011. UL46, a late gene of herpesvirus, codes for the abundant but non-essential viral phosphoproteins 11 and 12 (VP11/12). In this study, VP11/12 was found to localize inside both the nucleus and cytoplasm. The nuclear localization signal (NLS) of VP11/12 was identified as ^3^RRARGTRRASWKDASR^18^. Further research identified α5 and α7 to be the receptors for NLS and the chromosome region maintenance 1 (CRM1) to be the receptor for the nuclear export signal. Moreover, we found that PRV VP11/12 interacts with EP0 and the stimulator of interferon genes protein (STING), whereas the NLS of VP11/12 is the important part for VP11/12 to interact with UL48. To our knowledge, this is the first study to provide reliable evidence verifying the nuclear localization of VP11/12 and its role as an additional shuttling tegument protein for PRV. In addition, this is also the first study to elucidate the interactions between PRV VP11/12 and EP0 as well as between PRV VP11/12 and STING, while identifying the precise interaction sites of PRV VP11/12 and VP16.

## Introduction

Pseudorabies virus (PRV) is the pathogen for pseudorabies (PR), which is associated with nervous system disorders, respiratory disorders, and reproductive failure, resulting in massive economic loss to the swine industry in China, especially owing to the PRV variant that has emerged since 2011 (1–3).

The taxonomic name for PRV is Suid herpesvirus 1, whereas its original name is Aujeszky's disease virus. PRV is a member of the genus *Varicellovirus* in the subfamily Alphaherpesvirinae, which belongs to the family Herpesviridae. According to a previous study, all PRV genes contain homologs in at least one related alpha herpesvirus. Thus, most genes and certain protein names are derived from their locations within unique regions, in conformity with the prototypical herpes simplex virus type 1 (HSV-1) (4). The structure of the PRV mature virion consists of a linear dsDNA genome, a capsid, a tegument (a protein matrix), and an envelope (4, 5). The PRV genome is primarily characterized by a unique short (US) region, a unique long (UL) region, internal repeat sequences (IRS), and terminal repeat sequences (6).

The UL46 gene, a late gene of herpesvirus, encoding the abundant but non-essential viral phosphoproteins 11 and 12 (VP11/12) (7–9), is located within the UL regions of the viral genome. VP11/12 is one of the major tegument proteins with a molecular mass of ~95 kDa (10). The absence of UL46 in the HSV and PRV genomes was reported to have no serious impact on viral titers, one-step growth kinetics, virus replication, or virion morphogenesis in porcine kidney cells (PK-15 cells) and rabbit kidney cells (RK13 cells), only leading to a slight reduction of plaque size (10, 11). Furthermore, Thomas C. Mettenleiter demonstrated that in mice, the neuroinvasion and neurovirulence of PRV-ΔUL46 do not differ significantly from those of the classic PRV strain, Kaplan, via measurement of the mean survival times and viral titers in epithelial cells of nasal mucosa, first-order trigeminal neurons, second-order trigeminal neurons, and pons of infected mice, using immunohistochemistry (12).

However, UL46 is an important gene in herpesviruses and plays an important role in virion assembly and virus growth, especially in the secondary envelopment of viruses and in the inhibition of innate immunity upon viral infection. Moreover, VP11/12 has been reported to serve as the major component of virion tegument, in the form of conserved clusters and functional complexes with pUL47, pUL48, and pUL49 in HSV and PRV (13–15). Additionally, in a study seeking to examine the virus/virus or virus/host interactions, HSV-1 was used to infect human fibroblasts, and VP11/12-related proteins were found as complexes, by immune-affinity purification and mass spectrometry. Further, in this study, at least 23 sites of VP11/12 were shown to be phosphorylated (16). In T cells and primary fibroblasts infected with HSV-1, VP11/12 was shown to function as a substrate of Lck or a Lck-activating kinase and to participate in PI3K-Akt signaling involving virus-induced activation (17–19). In PRV, VP11/12 can induce phosphorylation and expression of ERK1/2; however, it fails to activate the PI3K-Akt signaling pathway.

In previous studies, subcellular localization of VP11/12 was observed in the cytoplasmic or perinuclear regions, in both plasmid-transfected cells and virus-infected cells. In our study, we investigated the intrinsic properties of VP11/12, in the absence of other viral proteins or in PRV JS-2012 infection, and found that VP11/12 localized to the nucleus, as observed by indirect immunofluorescence, and gradually appeared in the cytoplasm as well. Therefore, we sought to elucidate the specific mechanisms and vital functions of the nuclear localization signal (NLS) and nuclear export signal (NES) of VP11/12.

## Materials and Methods

### Cell Lines and Viruses

Vero cells, HeLa cells, 293T cells, and PRV variant strain JS-2012 were obtained from the Shanghai Veterinary Research Institute, Chinese Academy of Agricultural Sciences (SHVRI-CAAS, Shanghai, China). Cos-7 cells and NIH/3T3 cells were kindly provided by the Shanghai Stem Cell Bank, Chinese Academy of Sciences (Shanghai, China). All cell lines were grown in Dulbecco's Modified Eagle Medium (DMEM, Gibco, USA) supplemented with 10% fetal bovine serum (FBS, Gibco) at 37°C.

### Antibodies and Reagents

Polyclonal antibody anti-UL46 was obtained from SHVRI-CAAS. Anti-FLAG and anti-HA monoclonal antibodies were purchased from Sigma-Aldrich (USA). DAPI and leptomycin B (LMB) were purchased from Beyotime Institute of Biotechnology (Shanghai, China). The monoclonal antibody anti-lamin B2 was purchased from Cell Signaling Technology (CST, USA). Donkey anti-mouse IgG secondary antibodies, Alexa Fluor 594 and Alexa Fluor 488, were purchased from Invitrogen (Thermo Fisher Scientific, USA). Anti-GFP monoclonal antibodies, HRP-conjugated goat anti-mouse IgG, and HRP-conjugated goat anti-rabbit IgG were purchased from ProteinTech (USA). All restriction endonucleases used for cloning procedures were purchased from New England Biolabs (NEB, USA), and homologous recombinases were procured from Vazyme Biotech Co. Ltd. (Nanjing, China). PrimeStar^®^ HS DNA Polymerase was purchased from TaKaRa (Japan).

### Plasmid Construction

The full-length UL46 open reading frame, including both the start codon methionine and the stop codon, was PCR-amplified from PRV strain JS-2012 (GenBank accession: KP257591) genomic DNA, using primers that incorporated *Eco*RI sites and homologous sequences in plasmids, p3xFlag-CMV, pEGFP-C3, or pCAGGS. The UL46 fragments were inserted into vectors, p3xFlag-CMV, pEGFP-C3, and pCAGGS, individually, to generate UL46-FLAG, UL46-EGFP, and UL46-HA, respectively. For the positive control, full-length UL47 of PRV JS-2012 genomic DNA was cloned into the *Eco*RI site of p3xFlag-CMV to generate UL47-FLAG. The vector pEGFP-C3 served as the negative control because it could express the enhanced green fluorescent protein (EGFP) protein.

The truncated and mutant UL46 fragments were cloned into the *Eco*RI site of the pEGFP-C3 vector to construct the required plasmids (Table S1). To generate plasmids expressing Flag-tagged α1, α3, α4, α5, α6, α7, and α8 were amplified from the cDNA of Vero cells and cloned into *Eco*RI sites of p3xFLAG-CMV. All fragments were inserted into the *Eco*RI site of pEGFP-C3. All primers are listed in Table S1. Each construct was confirmed by sequencing performed by the Shanghai Personal Biotechnology Limited Company, and no deletion, insertion, or mutation was detected.

### RNA Extraction and Reverse Transcription

Vero cells were collected and frozen at −80°C. Total RNA was extracted by a QIAGEN RNA Extraction Kit (QIAGEN, Dusseldorf, Germany) according to the manufacturer's instructions. Samples were then digested with DNase I to remove trace amounts of contaminating DNA. The integrity of total RNA was checked for cDNA synthesis using SuperScript™ III reverse transcriptase (Invitrogen, USA).

### Transfections and LMB Treatment

To express all proteins *in vitro*, a monolayer of Vero cells, HeLa cells, NIH/3T3 cells, or Cos-7 cells were plated overnight on coverslips in six-well plates (Corning, NY, USA) in DMEM (Gibco, USA) containing 10% FBS, at a density of 2 × 10^5^ cells per well, before transfection. Cells were transfected with mixtures of 1.5–2.0 μg plasmid DNA, Opti-MEM (Gibco, USA), and FuGENE HD Transfection Reagent (Promega, USA), according to the manufacturer's instructions. Indirect immunofluorescence assay (IFA) was then performed. For certain samples, LMB was added into the culture medium at a final concentration of 10 and 20 ng/ml for 4 h.

### Immunofluorescence and Confocal Microscopy

After transfection for 24 h, cells were fixed in ice-cold absolute methanol at −20°C for 30 min, washed three times in PBS, and blocked with PBS containing 5% bull serum albumin (Yeasen, Shanghai, China) for 1 h at 28°C. Subsequently, cells were washed three times with PBS and incubated with anti-Flag monoclonal antibody as the primary antibody in six-well plates for 1 h at 37°C. After washing with PBS three times, donkey anti-rabbit IgG secondary antibody, Alexa Fluor 594 (Invitrogen, Shanghai) in PBS, was added to plates for 1 h at 37°C. After each incubation step, cells were washed three times extensively with PBS and stained with DAPI (Beyotime Biotechnology). Samples were analyzed using a Zeiss LSM 880 laser scanning confocal microscope. Images were processed with Adobe Photoshop.

### Immunoprecipitation (IP) and Western Blotting Assays

These experiments were carried out as described previously (31); 293T cells were transfected alone or in combination with plasmids UL46-HA, α1-FLAG, α3-FLAG, α4-FLAG, and α6-FLAG using Lipofectamine 3000 (Invitrogen, USA). At 24-h post transfection (hpt), the cells were washed twice with cold PBS and lysed with IP lysis buffer (Thermo Fisher) mixed with inhibitors for protease and phosphatases (BioTool). Except for the inputs of lysates, other cell lysates were incubated with anti-HA agarose (Sigma, cat# A2095) at 4°C overnight. The immunoprecipitates were washed four times with IP lysis buffer and then subjected to western blotting analysis.

### Statistical Analysis

Data are presented as mean ± SD. Statistical analysis was performed using GraphPad Prism version 7.0 (San Diego, CA, USA). Differences were considered statistically significant at *P* < 0.05.

## Results

### VP11/12 Localized in the Nucleus and Cytoplasm in the Presence or Absence of Other Viral Proteins

**Figure 1:**
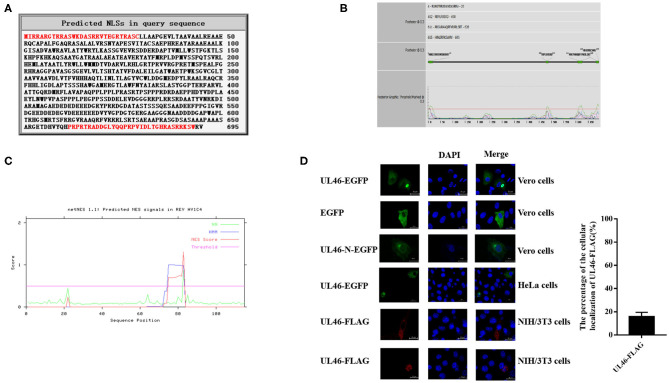
pUL46 as a nucleocytoplasmic shuttling protein. **(A)** cNLS Mapper prediction of the NLSs of pUL46 (in red). **(B)** NLStradamus prediction of the NLSs of pUL46 (in green squares). **(C)** NetNES prediction of the NESs of pUL46 (the peaks of lines). **(D)** Vero cells were transfected with UL46-EGFP, pEGFP-C3, and UL46-N-EGFP; HeLa cells, UL46-EGFP; and NIH/3T3 cells, UL46-FLAG. After 24 h of transfection, Vero, HeLa, and NIH/3T3 cells were subjected to immunofluorescence analysis. Scale bars = 20 μm.

To study the functions of VP11/12, the NLSs of VP11/12 were predicted via cNLS Mapper (20, 21) and NLStradamus (22). The cNLS Mapper found two potential NLSs in the arginine-rich regions of VP11/12, namely, ^2^IRRARGTRRASWKDASRRVTEGRTRASC^29^ and ^662^PRPRTRADDGLYQQPRPVIDLTGHRASRRKSW^693^ (Figure 1A). NLStradamus also revealed four potential NLS motifs in VP11/12: ^4^RARGTRRASWKDASRRV^20^, ^482^RRPLRRSRD^490^, ^611^RRGVRAAQRFVRRRLSRT^628^, and ^685^HRASRRKSWRV^695^ (Figure 1B). The NESs of VP11/12 were predicted by NetNES (23), showing that the N-terminal 22nd or 73rd−83rd sequences might have NES (Figure 1C). Thus, we hypothesized that VP11/12 might localize in both the nucleus and cytoplasm, even though previous studies reported only a cytoplastic localization of VP11/12.

To identify the subcellular localization of VP11/12 in transfected cells in the absence of other viral proteins, the UL46 gene of the PRV variant JS-2012 was cloned into the C-terminus of EGFP in the eukaryotic expression vector pEGFP-C3 (UL46-EGFP) and in the C-terminus of FLAG in the p3xFlag-CMV vector (UL46-FLAG). The plasmid DNA of UL46-EGFP was transfected into live Vero cells and HeLa cells; 24 h later, cells were fixed with cold methanol. Alternatively, the plasmid of the UL46-FLAG was transfected into NIH/3T3 cells. After 24 h, they were fixed with cold methanol and washed three times with PBS. They were incubated with anti-flag monoclonal antibodies for 1 h at 37°C and then washed three times with PBS. The cells were then incubated with anti-mouse IgG secondary antibody Alexa Fluor 594 for 1 h at 37°C. In order to differentiate between nuclei and cytoplasm, all cells were stained with DAPI and assessed by a Zeiss LSM 880 laser scanning confocal microscope. Consequently, as shown in Figure 1D, the transfected UL46-EGFP displayed approximately 84% cytoplasmic and 16% nuclear localization in Vero cells; UL46-FLAG was also shown to be localized to the nuclei and cytoplasm of NIH/3T3 cells. To identify whether GFP in the C-terminus or N-terminus of UL46 could change the subcellular localization of VP11/12, we constructed a plasmid with GFP in the C-terminus of VP11/12, UL46-N-EGFP, and performed IFA analysis. Results of confocal microscopy showed that UL46-N-EGFP localized in both the nucleus and cytoplasm. This indicated that the location of EGFP in either the C-terminus or N-terminus did not influence the subcellular localization of VP11/12; therefore, in subsequent experiments, we chose EGFP in the N-terminus of VP11/12, allowing VP11/12 to translocate to both the nucleus and cytoplasm.

**Figure 2:**
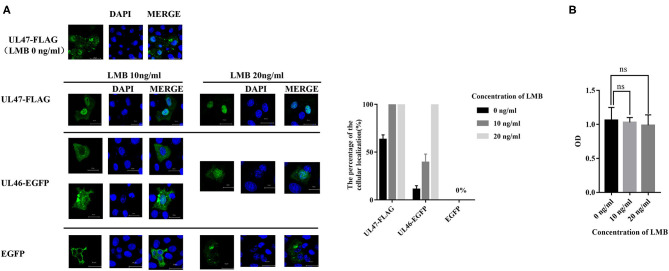
Nuclear localization of UL46-EGFP in cells treated with LMB. **(A)** Vero cells were transfected with UL47-FLAG (positive control), UL46-EGFP, and EGFP-C3 (negative control) and treated with LMB for 24 h. All cell nuclei were stained with DAPI and observed using a laser confocal microscope. Scale bars = 20 μm. **(B)** Cellular toxicity of Vero cells treated with LMB was analyzed using CCK-8 assays.

A previous study revealed LMB is an inhibitor of CRM1, one of the major nuclear export receptors for leucine-rich NES-containing proteins (24). We analyzed the amino acid sequence of VP11/12 and concluded it to be a leucine-rich protein, with 53 leucine residues out of 695 amino acids. Based on the prediction for NESs of VP11/12, we hypothesized that CRM1 would be the export receptor. To support our hypothesis, Vero cells were transfected with plasmids of UL46-EGFP and treated with 10 or 20 ng/ml of LMB. The positive control, plasmid UL47-FLAG of PRV, was a proven shuttling protein (25), and the negative control, pEGFP-C3, was used in a similar manner as the treatment plasmids, followed by direct immunofluorescence. The results demonstrated that VP11/12 partially localized to the nucleus when cells were treated with 10 ng/ml of LMB and completely localized to the nucleus in the presence of 20 ng/ml, whereas pUL47 alone remained in the nucleus and EGFP localized to the cytoplasm in cells treated with 10 or 20 ng/ml of LMB (Figure 2). These results indicated that VP11/12 covalently modifies CRM1 for export to the cytoplasm, depending on the LMB concentration.

**Figure 3:**
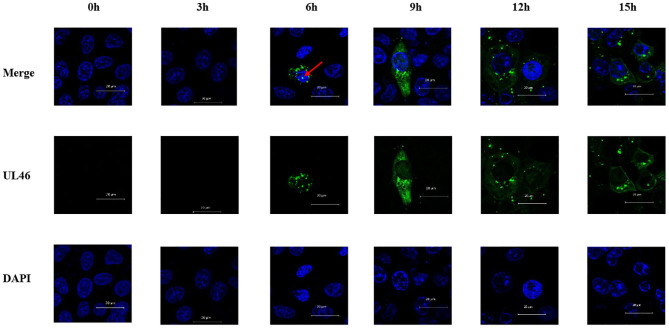
Nuclear localization of VP11/12 during PRV infection. Vero cells were infected with PRV JS-2012 for 0, 3, 6, 9, 12, and 15 h at a multiplicity of infection of 0.01. Infected cells were subjected to immunofluorescence analysis with anti-pUL46 polyclonal antibody, and the nuclei were stained with DAPI. Scale bar = 20 μm.

VP11/12 has been reported to be localized in the cytoplasm of HSV-1 and HSV-2. Michael Murphy et al. (26) hypothesized that removal of the N-terminal 446 amino acids from the HSV-1 VP11/12 would allow for transport of the protein into the nucleus, although no data were presented to support this hypothesis. In this study, Vero cells were seeded overnight into six-well plates with slides. Cells were infected with PRV variant strain JS-2012 at a multiplicity of infection (MOI) of 0.1 and were examined via IFA using an anti-UL46 polyclonal antibody and DAPI at 0, 3, 6, 9, 12, and 15 h post infection. All samples were observed by Zeiss confocal laser microscopy. IFA results revealed that VP11/12 was present in the nucleus after 6 h of PRV infection, although at a low rate (Figure 3).

All the above results demonstrate that VP11/12 translocates into the nucleus from the cytoplasm and is transported into the cytoplasm from the nucleus whether viral proteins were present or not. Moreover, CRM1 was identified as the nuclear export receptor for VP11/12.

### Mapping the NLS in VP11/12

**Figure 4:**
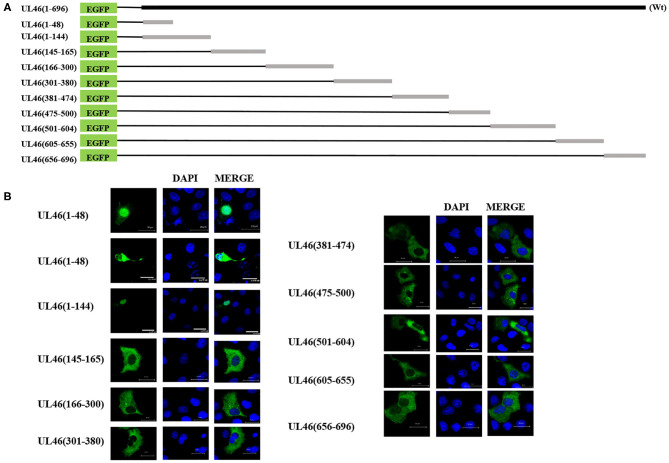
aa1–48 of UL46 as the functional NLS. **(A)** A schematic representation of truncated UL46 predicted by NLStradamus, fused with the C-terminus of EGFP. **(B)** Subcellular localization of the UL46 mutants in **(A)**. Scale bars = 20 μm.

Because of UL46 protein, approximately 100 kDa, UL46-EGFP was unable to enter the nucleus without the assistance of additional proteins (26, 27), unless one or more NLSs were present. Considering that some NLSs are short peptides, we constructed truncated fusion plasmids of UL46-EGFP (Figure 4A), based on the prediction of NLStradamus, and transfected them into Vero cells. After 24 h, subcellular distribution of the fusion proteins was assessed by indirect fluorescence using a Zeiss LSM 880 confocal microscope [UL46(145–165)-EGFP, UL46(166–300)-EGFP, UL46(301–380)-EGFP, UL46(381–474)-EGFP, UL46(475–500)-EGFP, UL46(501–604)-EGFP, UL46(605–655)-EGFP, and UL46(656–696)-EGFP] and Nikon C1-Si confocal microscope [UL46(1–48)-EGFP and UL46(1–144)-EGFP]. Strikingly, the N-terminal 48 amino acid residues from VP11/12 fusion with EGFP revealed nuclear accumulation (Figure 4B). Specifically, UL46(1–48)-EGFP and UL46(1–144)-EGFP accumulated not only in the nucleus but also in the cytoplasm. These results suggest the existence of an NLS and potentially an NES within the 48 N-terminal residues of VP11/12, thereby verifying that the NLS is located in the N-terminal amino acids (1–144) of VP11/12.

### Identification of the Precise NLS in VP11/12

**Figure 5:**
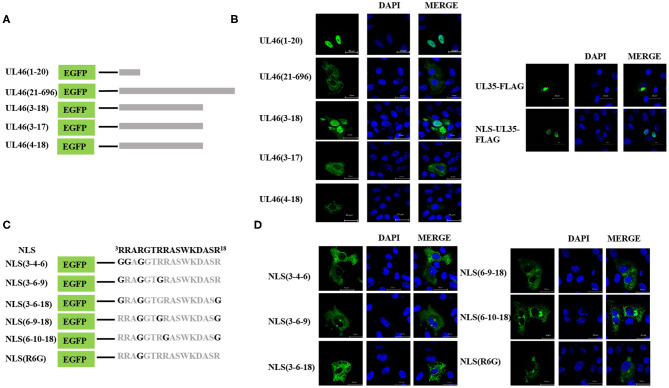
Precise identification of the NLS of UL46. **(A)** A schematic representation of deletion mutants aa1–20, aa21–696, aa3–18, aa3–17, and aa4–18 fused with the C-terminus of EGFP. **(B)** Localization of aa1–20, aa21–696, aa3–18, aa3–17, and aa4–18 mutants; NLS-UL35-FLAG; and UL35-FLAG. All cell nuclei were stained with DAPI. Scale bars = 20 μm. **(C)** Schematic representations of point mutations in the NLS. **(D)** Subcellular localization of proteins harboring point mutations in their NLSs. Scale bars = 20 μm.

To identify the NLS, the N-terminal 48-residue peptide was truncated, and the following related plasmids were generated: UL46(1–40)-EGFP, UL46(1–30)-EGFP, UL46(1–25)-EGFP, and UL46(1–20)-EGFP (Figure 5A). IFA revealed that all peptides were located in the nucleus (Figure 5B and data not shown).

Moreover, to confirm whether VP11/12 contains other NLSs, a plasmid with deletion of the N-terminal 1–20 amino acids, UL46(21–696), was constructed and transfected into Vero cells in six-well plates. UL46(21–696) was not localized in the nucleus but rather was distributed in the cytoplasm (Figure 5B). Furthermore, classical NLSs are known to be rich in arginine (28), and this classical characteristic was not observed in UL46(21–696). Hence, these results indicated that no additional classical NLS was present in VP11/12; rather the 20 N-terminal residues formed the only NLS.

To precisely locate the NLS of VP11/12, amino acid residues 18, 17, and 16 of UL46(1–20) were cloned into EGFP-C3, thereby constructing a series of deletions of UL46 (Figure 5A). Subcellular localization of these mutants was analyzed, and UL46(3–18)-EGFP was found to display nuclear localization, whereas UL46(3–17)-EGFP, UL46(4–18)-EGFP, and other mutants were only present in the cytoplasm (Figure 5B and data not shown). Based on the above evidence, the ^3^RRARGTRRASWKDASR^18^ peptide was considered to have sufficient residues to guide VP11/12 to the nucleus. To further ensure the effectiveness of the UL46(3–18), we constructed the plasmid NLS-UL35-FLAG fusing with the NLS and PRV UL35 and UL35-FLAG. PRV VP26 encoded by gene UL35 was verified only by cytoplasmic localization without other PRV viral proteins (29, 30). The results showed that all NLS-UL35-FLAG translocated into the nuclei while UL35-FLAG could not. Therefore, it could be concluded that the NLS of UL46 facilitated the transport of NLS-UL35-FLAG into the nuclei. Hence, ^3^RRARGTRRASWKDASR^18^ was confirmed as the NLS of VP11/12.

Nuclear localization signals consist of many basic amino acids, especially arginine (31). There are 6 arginine residues in UL46(3–18). To identify the critical amino acids in the NLS of VP11/12, glycine replacement mutagenesis was performed with respect to the wide-type UL46(3–18) (Figure 5C). Immunofluorescence assay results using the mutants were compared to those of wide type UL46(3–18). All mutants, including NLS (3-4-6), NLS (3-6-9), NLS (3-6-18), NLS (6-9-18), and NLS (6-10-18), were present in the cytoplasm, and were absent from the nucleus (Figure 5D). Coincidently, among these mutants, the sixth amino acid had been changed from arginine to glycine. Therefore, we only mutated the sixth amino acid to glycine in UL46(3–18) to generate NLS (R6G). Confocal microscopy results revealed that NLS (R6G) localized in the cytoplasm (Figure 5D). Thus, UL46(3–18) peptide was identified as the NLS of VP11/12 and the sixth N-terminal amino acid-arginine in UL46(3–18) was found to be the key element that determines the subcellular localization of VP11/12.

### Binding of VP11/12 to α5 and α7 via NLS

**Figure 6:**
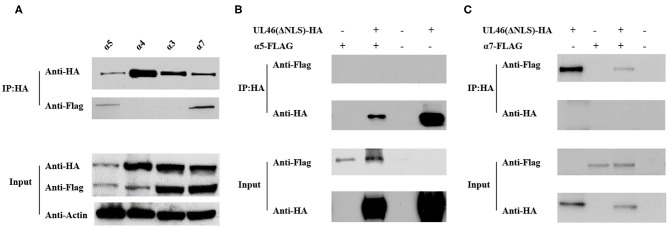
UL46 binds to importins. **(A)** IP for 293T cells co-transfected with recombinant constructs encoding UL46-HA and FLAG-tagged α3, α4, α5, and α7. **(B)** IP for 293T cells co-transfected with recombinant constructs encoding UL46-ΔNLS-HA and α5. **(C)** IP for 293T cells co-transfected with recombinant constructs encoding UL46-ΔNLS-HA and α7.

The results described above indicate that VP11/12 contains a classical NLS, which may direct it to the nucleus. Thus, we considered the importin α/β pathway as a mediator of VP11/12 nuclear import. Seven different isoforms of importin have been characterized in mammalian cells (32, 33). To confirm our hypothesis, we constructed FLAG-tagged α1, α3, α4, α5, α6, α7, and α8 co-transfected with UL46-HA or ΔNLS-HA into 293T cells and performed IP assays, according to the standard protocol (34). After co-transfection, 293T cells were lysed by Pierce IP lysis buffer using protease inhibitors, and the lysates of the co-transfected cell were incubated with HA-tagged beads (Sigma, USA) overnight at 4°C. Thereafter, SDS-PAGE and western blotting were performed for the samples. IP assay results showed α5 and α7 to be co-immunoprecipitated with UL46-HA. The IP samples were immunoblotted with anti-HA and anti-FLAG MAb (Sigma). α1, α3, α4, α7, and α8 were detected only in the input samples (data not shown). UL46-HA was verified in both the input and IP samples, which confirmed that UL46 did not interact with the importins α1-, α3-, α4-, α7-, and α8-FLAG (Figure 6 and data not shown). Furthermore, α5- and α7-FLAG were detected in both input and IP samples.

To verify that the NLS of VP11/12 is the interaction site between VP11/12 and α5 or α7, we constructed a plasmid, ΔNLS-UL46, in which the NLS of VP11/12 was truncated and contained the vector HA-tagged PCAGGS. The ΔNLS-UL46 was co-immunoprecipitated with α5- or α7-FLAG by HA-tagged beads. The results showed that α5 and α7 were detected in the input samples but not in the IP samples. These data revealed that α5 or α7 was possibly required for guiding VP11/12 to the nucleus and that deletion of NLS from VP11/12 abolished the VP11/12 binding to α5- or α7-FLAG. This further indicated that the α5 and α7 might meditate the nuclear import of VP11/12 (Figure 6).

### Binding of VP11/12 to UL48 Through the NLS of VP11/12

**Figure 7:**
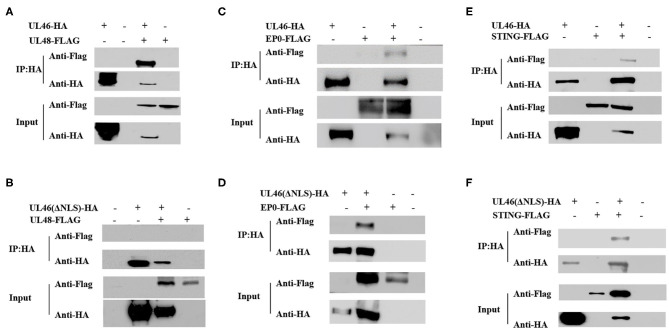
The NLS of UL46 interacts with UL48. **(A)** IP for 293T cells co-transfected with recombinant constructs encoding UL46-HA and UL48-FLAG. **(B)** IP for 293T cells co-transfected with recombinant constructs encoding UL46-ΔNLS-HA and UL48-FLAG. **(C)** IP for 293T cells co-transfected with recombinant constructs encoding UL46-HA and EP0-FLAG. **(D)** IP for 293T cells co-transfected with recombinant constructs encoding UL46-ΔNLS-HA and EP0-FLAG. **(E)** IP for 293T cells co-transfected with recombinant constructs encoding UL46-HA and STING-FLAG. **(F)** IP for 293T cells co-transfected with recombinant constructs encoding UL46-ΔNLS-HA and STING-FLAG.

UL48, encoding VP16, plays vital roles in promoting the assembly of a multicomponent complex with Oct-1 and HCF-1, to induce the expression of viral immediate–early genes (35) and secondary envelopment in both HSV-1 and PRV (36–39), and it can enter the nucleus in HSV-1 (40–42) and PRV (37). VP11/12 has been reported to interact with UL48 (11), although the exact site has not yet been identified. To confirm whether the NLS of VP11/12 interacts with UL48, the plasmid UL48-FLAG was constructed, co-transfected with UL46-HA, and subjected to co-immunoprecipitation. The IP assay demonstrated that UL48-FLAG was detected in the input as well as IP samples. Alternatively, UL48-FLAG could not be tested when ΔNLS-UL46-HA (not UL46-HA) was in the proteins. Therefore, the NLS of VP11/12 was likely the same domain used to allow VP11/12 to interact with pUL48, thereby regulating the functions of pUL48 (Figures 7A,B). These results suggest that the NLS of VP11/12 is the domain by which VP11/12 regulates the functions of UL48.

### Binding of VP11/12 to EP0 or STING Does Not Occur Through the NLS

EP0, the homolog of ICP0 in HSV-1, is a transactivating protein that can enhance the infectivity of PRV genomic DNA, couples with IE180 (43–45), and localizes to the nucleus in HSV-1 (42) and PRV (46). In HSV-1, the homolog of EP0, ICP0, interacts with VP11/12 (16, 17). To determine whether EP0 interacts with VP11/12 in PRV and whether the NLS of VP11/12 is the key element for VP11/12 to bind to EP0, we constructed the FLAG-tagged EP0 plasmid, EP0-FLAG. UL46-HA and ΔNLS-UL46 were co-transfected with EP0-FLAG, individually, in 293T cells, and the transfected cells were harvested after 30 hpt and subjected to co-immunoprecipitation. Results of SDS-PAGE and western blot analysis revealed that EP0-FLAG was expressed irrespective of co-transfection with UL46-HA or ΔNLS-UL46 (Figures 7C,D). Thus, the NLS of UL46 was not the key element.

HSV-1 VP11/12 has been reported to interact with STING via its 100 N-terminal amino acids (19). Therefore, we speculated that the N-terminus of PRV VP11/12 might also interact with STING. The IP assay in UL46-HA/STING-FLAG and ΔNLS-UL46/STING-FLAG showed that UL46-HA and ΔNLS-UL46 interacted with STING (Figures 7E,F). Therefore, the NLS was not the site contributing to their binding.

## Discussion

HSV-1 VP11/12 is a multifunctional tegument protein that interacts with many viral proteins and cellular proteins in immune signaling pathways, thereby contributing to several biological processes (17–19, 47–49). Moreover, HSV-1 VP11/12 and its homolog PRV VP11/12 both play roles in virus growth, most notably in the secondary envelopment (26, 27, 37). Previous studies on HSV-1 and PRV VP11/12 showed VP11/12 to be localized in the cytoplasm, rather than in the nucleus. Therefore, when nuclear localization of VP11/12 was observed, we considered determining its mechanism of nuclear localization, identifying the transport mechanisms, and investigating the significance of VP11/12 localization into the nucleus.

Nucleocytoplasmic shuttling proteins contain NLSs and NESs. Herein, we demonstrated that PRV VP11/12 shuttles between the nucleus and cytoplasm, which was the first report on nucleocytoplasmic shuttling of herpesvirus VP11/12. We also found that VP11/12 was to possibly localize in the nucleus and performed related experiments to identify the NLS. Furthermore, we confirmed that the nuclear export of VP11/12 occurred through its interaction with CRM1, because 20 ng/ml of LMB inhibited its nuclear export.

As schematically represented in Figure 4, a newly characterized NLS, containing basic clusters, was found to be responsible for the nuclear localization of VP11/12. After a series of mutation analyses and fluorescence microscopy, ^3^RRARGTRRASWKDASR^18^ was identified as the functional NLS. VP11/12 appeared to have been phosphorylated heavily in at least 23 sites when HSV-1 infected, and phosphorylation played important roles in modulating VP11/12 abundance with respect to ICP0 localization and interactions (16). According to the alignment of specific herpesvirus VP11/12 protein sequences, there was likely no phosphorylated site in the PRV NLS of VP11/12 (data not shown).

To identify the NLS of VP11/12, we truncated 48 amino acids from the N-terminus of VP11/12 and generated the plasmid UL46(1–48)-EGFP. Subsequent experiments demonstrated that UL46(1–48)-EGFP could be seen in both the nucleus and the cytoplasm (Figure 4B). Therefore, it was reasonably hypothesized that there is an NES in the N-terminal 48-amino-acid stretch. Considering that the NLS of VP11/12 is located in the N-terminal 3rd to 18th amino acids, coupled with previous studies, the export of leucine-rich NES-containing proteins may be inhibited by LMB (50); we predicted the NES to be between the 19th and 48th amino acids of VP11/12. To verify this hypothesis, we created point mutations containing leucine residues, generated the plasmid UL46(L30G-L31G-L38G-L45G)-EGFP, and observed it with a confocal microscope. Our result showed that UL46(L30G-L31G-L38G-L45G)-EGFP appeared in both the cytoplasm and nucleus (data not shown), thus indicating that the efficient NES is not the classical one that is located between the 19th and 48th residues. Therefore, we speculated that the NES in the N-terminus of VP11/12 may not be classical; and further experiments needs to be conducted to test for a more functional NES.

In a previous study, HSV-1 VP11/12 had been proven to interact with the host protein STING (19) and the viral protein ICP0, and HSV-1 and PRV VP11/12 interacted with UL48 (11); however, the interaction sites of VP11/12 were not all localized. Therefore, in this study, we proved for the first time that PRV VP11/12 interacted with the crucial antiviral protein STING, whereas ΔNLS-UL46 also interacted with STING, thus demonstrating that in PRV, VP11/12 could regulate the functions of STING in the cytoplasm and help PRV DNA to evade the host innate immunity (19). Furthermore, it is the first time to show that PRV VP11/12 interacts with PRV EP0, similar to that with STING and that ΔNLS-UL46 also interacts with EP0, thus possibly indicating that in PRV, EP0 helps to maintain the proper expression level of VP11/12. Considering that PRV EP0 and its homologous protein HSV-1 ICP0 can be transported between the cytoplasm and nucleus (46, 51) and that ICP0 influences numerous host nuclear proteins, such as cyclin D3, p53, ND10, p60, and the ubiquitin-specific protease USP7, in addition to regulating viral gene expression, stimulating lytic infection, and enhancing reactivation of latent infection (52–57), we reasonably assumed that VP11/12 might exhibit connections with the functions of EP0 in the nucleus.

The PRV VP11/12 interacted with PRV pUL48, and more importantly, the NLS of VP11/12 interacted with pUL48/VP16, the key activator of lytic infection by initiating the lytic program (37). A previous study revealed the translocation of pUL48 into the nucleus, depending on HCF (also referred to as C1, CFF, or VCAF) owing to the absence of NLS (58). In the late phase of HSV-2 infection, most VP11/12 co-localized with VP16, and VP11/12 could enhance α4 promoter-regulated gene expression when co-precipitating with VP16 (27). Thus, we hypothesized that the NLS of PRV VP11/12 was conducive to the activation of PRV induced by pUL48, even though VP11/12 was not essential.

In summary, we verified the nucleocytoplasmic shuttling of VP11/12 and identified its NLS, ^3^RRARGTRRASWKDASR^18^. Furthermore, we identified interactions between PRV VP11/12 and EP0, PRV VP11/12 and pUL48, and PRV VP11/12 and the host protein, STING, which is critical for the growth of PRV. The significance of these interactions requires further investigation.

## Data Availability Statement

The raw data supporting the conclusions of this article will be made available by the authors, without undue reservation.

## Author Contributions

JX: experiment, writing, and design. FG: supervise and revise. JW: experiment and design. HZhe: supervise and design. XC, YL, HZhu, and XF: experiment. WT, YJ, LL, and NK: supervise. GL and GT: funding. All authors contributed to the article and approved the submitted version.

## Conflict of Interest

The authors declare that the research was conducted in the absence of any commercial or financial relationships that could be construed as a potential conflict of interest.

## Abbreviations

CRM1: chromosome region maintenance 1
DAPI, 4′: 6-diamidino-2-phenylindole
EGFP: enhanced green fluorescent protein
HSV-1: herpes simplex virus type 1
HSV-2: herpes simplex virus type 2
IFA: immunofluorescence assay
LMB: leptomycin B
NES: nuclear export signal
NLS: nuclear localization signal
PRV: pseudorabies virus
STING: stimulator of interferon genes protein
TRS: terminal repeat sequence
UL: unique long regions
US: unique short regions.
