# Supplementary material for: Characterization of Nucleocytoplasmic Shuttling of Pseudorabies Virus Protein UL46
Source: Front Vet Sci. 2020 Aug 21;7:484. doi: 10.3389/fvets.2020.00484 (PMC7472561; doi:10.3389/fvets.2020.00484)
Supplement: Supplementary file 1 [file Table_1.DOCX]

**Supplementary Materials**

**Table S1. Primers for the truncated protein pUL46 and mentioned proteins.**

| Primers | Sequences(5’-3’) |
| --- | --- |
| UL46-FLAG F  UL46-FLAG R | atcagatctatcgatgaattTCACACGCGCCAGCTCTT  atcagatctatcgatgaattTCACACGCGCCAGCTCTT |
| UL46-EGFP F  UL46-EGFP R | tcgagctcaagcttcgaattctgATGATCCGCCGCGCCCGA  gtaccgtcgactgcagaattTCACACGCGCCAGCTCTT |
| UL46(1-144) F  UL46(1-144) R | tcgagctcaagcttcgaattctgATGATCCGCCGCGCCCGA  gtaccgtcgactgcagaattCGTCGACCACAGCAGCATG |
| UL46(145-165) F  UL46(145-165) R | tcgagctcaagcttcgaattctgTTCGGCAAGACGCTGTCGAAGCACCCGTTCAAGCACAAGGCC  gtaccgtcgactgcagaattGGCGCCGTACGCGGCGCTCTGGGCCTTGTGCTTGAACGGGTGCTTCGACAGCGTCTTGCCGAA |
| UL46(166-300) F  UL46(166-300) R | tcgagctcaagcttcgaattctgACGCGCGCCGCGCTCGCC  gtaccgtcgactgcagaattCGTGAGGCCGCACACGCC |
| UL46(301-380) F  UL46(301-380) R | tcgagctcaagcttcgaattctgGCCGCCGTCGTGGCCGCC  gtaccgtcgactgcagaattGTAGTTGAACCAGGCGAGCG |
| UL46(381-474) F  UL46(381-474) R | tcgagctcaagcttcgaattctgGCCATCGCGCGCAGCCTG  gtaccgtcgactgcagaattCTCGTCGTCCGAGCTCGG |
| UL46(475-500) F  UL46(475-500) R | cgagctcaagcttcgaattctgCTGGAGGTGGACGGCGGCGGGCGGCGCCCGCTCCGGCGCAGCCGCGACGCCGCCACGTACGTGAACCGCAAGGACATT  gtaccgtcgactgcagaattAATGTCCTTGCGGTTCACGTACGTGGCGGCGTCGCGGCTGCGCCGGAGCGGGCGCCGCCCGCCGCCGTCCACCTCCAG |
| UL46(501-604) F  UL46(501-604) R | tcgagctcaagcttcgaattctgGCCCGCGCGATGGCGGGG  gtaccgtcgactgcagaattCCCGTGGCGCGTCAGCGG |
| UL46(605-655) F  UL46(605-655) R | tcgagctcaagcttcgaattctgAGCATGCGCACCAGCTTC  gtaccgtcgactgcagaattGGTCTCGCCGCGGGCGGA |
| UL46(656-696) F  UL46(656-696) R | tcgagctcaagcttcgaattctgGACCACGTGTACCAGCACCC  gtaccgtcgactgcagaattTCACACGCGCCAGCTCTT |
| UL46(1-48) R  UL46(1-48) R | tcgagctcaagcttcgaattctgATGATCCGCCGCGCCCGA  gtaccgtcgactgcagaattCGCCTCGCGCAGGGCCGC |
| UL46(49-96) F  UL46(49-96) R | tcgagctcaagcttcgaattctgGCCGAGCGCCAGTGCGCG  gtaccgtcgactgcagaattCTCGGCCGCGCGCGCGTA |
| UL(97-144) F  UL46(1-144) R | tcgagctcaagcttcgaattctgGCCGCGCTGAAGGGCATC  gtaccgtcgactgcagaattCGTCGACCACAGCAGCATG |
| UL46 1-20 F  UL46 1-20 R | tcgagctcaagcttcgaattctgATCCGCCGCGCCCGAGGA  gtaccgtcgactgcagaattGACGCGGCGCGAGGCATC |
| UL46 21-48 F  UL46 21-48 R | tcgagctcaagcttcgaattctgACCGAGGGGCGCACCCGC  gtaccgtcgactgcagaattCGCCTCGCGCAGGGCCGC |
| UL46 1-25 F  UL46 1-25 R | tcgagctcaagcttcgaattctgATCCGCCGCGCCCGAGGA  gtaccgtcgactgcagaattGGTGCGCCCCTCGGTGAC |
| UL46 1-30 F  UL46 1-30 R | tcgagctcaagcttcgaattctgATCCGCCGCGCCCGAGGA  gtaccgtcgactgcagaattCAGGCAGCTGGCGCGGGT |
| UL46 1-40 F  UL46 1-40 R | tcgagctcaagcttcgaattctgATCCGCCGCGCCCGAGGA  gtaccgtcgactgcagaattCGCCGTCAGCACCTCCCC |
| UL46 2-6 F  UL46 2-6 R | tcgagctcaagcttcgaattctgATCCGCCGCGCCCGA  gtaccgtcgactgcagaattTCGGGCGCGGCGGAT |
| UL46 2-12 F  UL46 2-12 R | tcgagctcaagcttcgaattctgATCCGCCGCGCCCGAGGAACGCGCCGCGCTTCG  gtaccgtcgactgcagaattCGAAGCGCGGCGCGTTCCTCGGGCGCGGCGGAT |
| UL46 3-10 F  UL46 3-10 R | tcgagctcaagcttcgaattctgCGCCGCGCCCGAGGAACGCGCCGC  gtaccgtcgactgcagaattGCGGCGCGTTCCTCGGGCGCGGCG |
| UL46 9-14 F  UL46 9-14 R | tcgagctcaagcttcgaattctgCGCCGCGCTTCGTGGAAG  gtaccgtcgactgcagaattCTTCCACGAAGCGCGGCG |
| UL46 12-20 F  UL46 12-20 R | tcgagctcaagcttcgaattctgTCGTGGAAGGATGCCTCGCGCCGCGTC  gtaccgtcgactgcagaattGACGCGGCGCGAGGCATCCTTCCACGA |
| UL46 14-20 F  UL46 14-20 R | tcgagctcaagcttcgaattctgAAGGATGCCTCGCGCCGCGTC  gtaccgtcgactgcagaattGACGCGGCGCGAGGCATCCTT |
| UL46 2-14 F  UL46 2-14 R | tcgagctcaagcttcgaattctgATCCGCCGCGCCCGAGGAACGCGCCGCGCTTCGTGGAAGGAT  gtaccgtcgactgcagaattATCCTTCCACGAAGCGCGGCGCGTTCCTCGGGCGCGGCGGAT |
| UL46 3-16 F  UL46 3-16 R | tcgagctcaagcttcgaattctgCGCCGCGCCCGAGGAACGCGCCGCGCTTCGTGGAAGGATGCC  gtaccgtcgactgcagaattGGCATCCTTCCACGAAGCGCGGCGCGTTCCTCGGGCGCGGCG |
| UL46 4-17 F  UL46 4-17 R | tcgagctcaagcttcgaattctgCGCGCCCGAGGAACGCGCCGCGCTTCGTGGAAGGATGCCTCG  gtaccgtcgactgcagaattCGAGGCATCCTTCCACGAAGCGCGGCGCGTTCCTCGGGCGCG |
| UL46 5-18 F  UL46 5-18 R | tcgagctcaagcttcgaattctgGCCCGAGGAACGCGCCGCGCTTCGTGGAAGGATGCCTCGCGC  gtaccgtcgactgcagaattGCGCGAGGCATCCTTCCACGAAGCGCGGCGCGTTCCTCGGGC |
| UL46 6-19 F  UL46 6-19 R | tcgagctcaagcttcgaattctgCGAGGAACGCGCCGCGCTTCGTGGAAGGATGCCTCGCGCCGC  gtaccgtcgactgcagaattGCGGCGCGAGGCATCCTTCCACGAAGCGCGGCGCGTTCCTCG |
| UL46 7-20 F  UL46 7-20 R | tcgagctcaagcttcgaattctgGGAACGCGCCGCGCTTCGTGGAAGGATGCCTCGCGCCGCGTC  gtaccgtcgactgcagaattGACGCGGCGCGAGGCATCCTTCCACGAAGCGCGGCGCGTTCC |
| UL46 2-17 F  UL46 2-17 R | tcgagctcaagcttcgaattctgATCCGCCGCGCCCGAGGAACGCGCCGCGCTTCGTGGAAGGATGCCTCG  gtaccgtcgactgcagaattCGAGGCATCCTTCCACGAAGCGCGGCGCGTTCCTCGGGCGCGGCGGAT |
| UL46 3-18 F  UL46 3-18 R | tcgagctcaagcttcgaattctgCGCCGCGCCCGAGGAACGCGCCGCGCTTCGTGGAAGGATGCCTCGCGC  gtaccgtcgactgcagaattGCGCGAGGCATCCTTCCACGAAGCGCGGCGCGTTCCTCGGGCGCGGCG |
| UL46 4-19 F  UL46 4-19 R | tcgagctcaagcttcgaattctgCGCGCCCGAGGAACGCGCCGCGCTTCGTGGAAGGATGCCTCGCGCCGC  gtaccgtcgactgcagaattGCGGCGCGAGGCATCCTTCCACGAAGCGCGGCGCGTTCCTCGGGCGCG |
| UL46 5-20 F  UL46 5-20 R | tcgagctcaagcttcgaattctgGCCCGAGGAACGCGCCGCGCTTCGTGGAAGGATGCCTCGCGCCGCGTC  gtaccgtcgactgcagaattGACGCGGCGCGAGGCATCCTTCCACGAAGCGCGGCGCGTTCCTCGGGC |
| UL46 2-16 F  UL46 2-16 R | tcgagctcaagcttcgaattctgATCCGCCGCGCCCGAGGAACGCGCCGCGCTTCGTGGAAGGATGCC  gtaccgtcgactgcagaattGGCATCCTTCCACGAAGCGCGGCGCGTTCCTCGGGCGCGGCGGAT |
| UL46 3-17 F  UL46 3-17 R | tcgagctcaagcttcgaattctgCGCCGCGCCCGAGGAACGCGCCGCGCTTCGTGGAAGGATGCCTCG  gtaccgtcgactgcagaattCGAGGCATCCTTCCACGAAGCGCGGCGCGTTCCTCGGGCGCGGCG |
| UL46 4-18 F  UL46 4-18 R | tcgagctcaagcttcgaattctgCGCGCCCGAGGAACGCGCCGCGCTTCGTGGAAGGATGCCTCGCGC  gtaccgtcgactgcagaattGCGCGAGGCATCCTTCCACGAAGCGCGGCGCGTTCCTCGGGCGCG |
| UL46 5-19 F  UL46 5-19 R | tcgagctcaagcttcgaattctgGCCCGAGGAACGCGCCGCGCTTCGTGGAAGGATGCCTCGCGCCGC  gtaccgtcgactgcagaattGCGGCGCGAGGCATCCTTCCACGAAGCGCGGCGCGTTCCTCGGGC |
| UL46 6-20 F  UL46 6-20 R | tcgagctcaagcttcgaattctgCGAGGAACGCGCCGCGCTTCGTGGAAGGATGCCTCGCGCCGCGTC  gtaccgtcgactgcagaattGACGCGGCGCGAGGCATCCTTCCACGAAGCGCGGCGCGTTCCTCG |
| UL46(2-18) F  UL46(2-18) R | tcgagctcaagcttcgaattctgATCCGCCGCGCCCGAGGAACGCGCCGCGCTTCGTGGAAGGATGCCTCGCGC  gtaccgtcgactgcagaattGCGCGAGGCATCCTTCCACGAAGCGCGGCGCGTTCCTCGGGCGCGGCGGAT |
| UL46(3-19) F  UL46(3-19) R | tcgagctcaagcttcgaattctgCGCCGCGCCCGAGGAACGCGCCGCGCTTCGTGGAAGGATGCCTCGCGCCGC  gtaccgtcgactgcagaattGCGGCGCGAGGCATCCTTCCACGAAGCGCGGCGCGTTCCTCGGGCGCGGCG |
| UL46(4-20) F  UL46(4-20) R | tcgagctcaagcttcgaattctgCGCGCCCGAGGAACGCGCCGCGCTTCGTGGAAGGATGCCTCGCGCCGCGTC  gtaccgtcgactgcagaattGACGCGGCGCGAGGCATCCTTCCACGAAGCGCGGCGCGTTCCTCGGGCGCG |
| UL(2-19) F  UL(2-19) R | tcgagctcaagcttcgaattctgATCCGCCGCGCCCGAGGAACGCGCCGCGCTTCGTGGAAGGATGCCTCGCGCCGC  gtaccgtcgactgcagaattGCGGCGCGAGGCATCCTTCCACGAAGCGCGGCGCGTTCCTCGGGCGCGGCGGAT |
| UL46(3-20) F  UL46(3-20) R | tcgagctcaagcttcgaattctgCGCCGCGCCCGAGGAACGCGCCGCGCTTCGTGGAAGGATGCCTCGCGCCGCGTC  gtaccgtcgactgcagaattGACGCGGCGCGAGGCATCCTTCCACGAAGCGCGGCGCGTTCCTCGGGCGCGGCG |
| UL46(2-20) F  UL46(2-20) R | tcgagctcaagcttcgaattctgATCCGCCGCGCCCGAGGAACGCGCCGCGCTTCGTGGAAGGATGCCTCGCGCCGCGTC  gtaccgtcgactgcagaattGACGCGGCGCGAGGCATCCTTCCACGAAGCGCGGCGCGTTCCTCGGGCGCGGCGGAT |
| 46-R3G-R4G-R6G F  46-R3G-R4G-R6G R | gaattctggGAgGAGCCgGAGGAACGCGCCGCGCTTCGTGGAAGGATGCCTCGCGCCGC  gcagaattGCGGCGCGAGGCATCCTTCCACGAAGCGCGGCGCGTTCCTCcGGCTCcTCc |
| 46-R3G-R6G-R9G F  46-R3G-R6G-R9G R | gaattctggGACGCGCGgGAGGAACGgGACGCGCTTCGTGGAAGGATGCCTCGCGCCGC  gcagaattGCGGCGCGAGGCATCCTTCCACGAAGCGCGTCcCGTTCCTCcCGCGCGTCc |
| 46-R3G-R6G-R18G F  46-R3G-R6G-R18G R | gaattctggGACGCGCGgGAGGAACGgGACGCGCTTCGTGGAAGGATGCCTCGgGACGG  gcagaattCCGTCcCGAGGCATCCTTCCACGAAGCGCGTCcCGTTCCTCcCGCGCGTCc |
| 46-R6G-R9G-R18G F  46-R6G-R9G-R18G R | gaattctgCGCCGCGCCgGAGGAACGgGACGCGCTTCGTGGAAGGATGCCTCGgGACGC  gcagaattGCGTCcCGAGGCATCCTTCCACGAAGCGCGTCcCGTTCCTCcGGCGCGGCG |
| 46-R6G-R10G-R18G F  46-R6G-R10G-R18G R | gaattctgCGCCGCGCCgGAGGAACGCGCgGAGCTTCGTGGAAGGATGCCTCGgGACGC  gcagaattGCGTCcCGAGGCATCCTTCCACGAAGCTCcGCGCGTTCCTCcGGCGCGGCG |
| 46-3-4-9-10-18 F  46-3-4-9-10-18 R | gaattctggGAgGAGCCCGAGGAACGgGAgGAGCTTCGTGGAAGGATGCCTCGgGAGGATGCCTCGgGAgGA  gcagaattTCcCGAGGCATCCTTCCACGAAGCTCcTCcCGTTCCTCGGGCTCcTCc |
| α5-FLAG F  α5-FLAG R | caagcttgcggccgcgaattcaATGACCACCCCAGGAAAAGA  atcagatctatcgatgaattTCAAAGCTGGAAACCTT |
| α1-FLAG F  α1-FLAG R | caagcttgcggccgcgaattcaATGTCCACCAACGAGAATGC  atcagatctatcgatgaattCTAAAAGTTAAAGGTCCCAG |
| α4-FLAG F  α4-FLAG R | caagcttgcggccgcgaattcaATGGCCGAGAACCCCAGCTT  atcagatctatcgatgaattTTAAAAATTAAATTCTTT |
| α5-FLAG F  α5-FLAG R | caagcttgcggccgcgaattcaATGGCGGACAACGAGAAACT  atcagatctatcgatgaattCTAAAACTGGAACCCTTCTG |
| α6-FLAG F  α6-FLAG R | caagcttgcggccgcgaattcaATGGATGCCATGGCTAGTCC  atcagatctatcgatgaattTTAAAGTTGAAATCCATCCA |
| α7-FLAG F  α7-FLAG R | caagcttgcggccgcgaattcaATGGAGACCATGGCGAGCCC  atcagatctatcgatgaattTTATAGCTGGAAGCCCTCC |
| α8-FLAG F  α8-FLAG R | caagcttgcggccgcgaattcaATGCCGACCTTAGATGCTCC  atcagatctatcgatgaattCTATTTTTTTGCTAAGCATT |
| ΔNLS-UL46 F  ΔNLS-UL46 R | catcattttggcaaagaattcatgACCGAGGGGCGCACCCGCGCCAGC  gatggtaccagatctgaattcTCAAGCGTAGTCTGGGACGTCGTATGGGTACACGCGCCAGCTCTTGC |
| UL48-FLAG F  UL48-FLAG R | caagcttgcggccgcgaattcaATGCGCGACGAGGAGTGCGTGGTC  atcagatctatcgatgaattTCACATCTCAAACATGCGGTT |
| EP0-FLAG F  EP0-FLAG R | caagcttgcggccgcgaattcaATGGACTGCCCCATCTGCCTGGACG  atcagatctatcgatgaattTCAGTCGTCGTCCTGGGTGAG |
| STING-FLAG F  STING-FLAG R | caagcttgcggccgcgaattcaATGACCCGCTCCAGTCTGCATCCA  atcagatctatcgatgaattTCAGGAGAAATCCGTGCGGAGA |

Lower case letters are sequences in vectors; upper case letters are sequences in UL46.
